# Expression of 2′,3′-cyclic nucleotide 3′-phosphodiesterase (CNPase) and its roles in activated microglia *in vivo* and *in vitro*

**DOI:** 10.1186/s12974-014-0148-9

**Published:** 2014-08-23

**Authors:** Lijuan Yang, Enci Mary Kan, Jia Lu, Chunyun Wu, Eng-Ang Ling

**Affiliations:** Department of Pathology and Pathophysiology, Kunming Medical University, 1168 West Chunrong Road, Kunming, 650500 Yunnan P.R China; Defense Medical and Environmental Research Institute, DSO National Laboratories, 27 Medical Drive, Singapore, 117510 Singapore; Department of Anatomy and Histology/Embryology, 1168 West Chunrong Road, Kunming, 650500 Yunnan P.R China; Department of Anatomy, Yong Loo Lin School of Medicine, National University of Singapore, Singapore, 117597 Singapore

**Keywords:** CNPase, Activated microglia, Lipopolysaccharide, Inflammatory mediators, Anti-inflammation

## Abstract

**Background:**

We reported previously that amoeboid microglial cells in the postnatal rat brain expressed 2′,3′-cyclic nucleotide 3′-phosphodiesterase (CNPase) both *in vivo* and *in vitro*; however, the functional role of CNPase in microglia has remained uncertain. This study extended the investigation to determine CNPase expression in activated microglia derived from cell culture and animal models of brain injury with the objective to clarify its putative functions.

**Methods:**

Three-day-old Wistar rats were given an intraperitoneal injection of lipopolysaccharide to induce microglial activation, and the rats were killed at different time points. Along with this, primary cultured microglial cells were subjected to lipopolysaccharide treatment, and expression of CNPase was analyzed by real-time reverse transcription PCR and immunofluorescence. Additionally, siRNA transfection was employed to downregulate CNPase in BV-2 cells. Following this, inducible nitric oxide synthase, IL-1β and TNF-α were determined at mRNA and protein levels. Reactive oxygen species and nitric oxide were also assessed by flow cytometry and colorimetric assay, respectively. In parallel to this, CNPase expression in activated microglia was also investigated in adult rats subjected to fluid percussion injury as well as middle cerebral artery occlusion.

**Results:**

*In vivo*, CNPase immunofluorescence in activated microglia was markedly enhanced after lipopolysaccharide treatment. A similar feature was observed in the rat brain after fluid percussion injury and middle cerebral artery occlusion. *In vitro*, CNPase protein and mRNA expression was increased in primary microglia with lipopolysaccharide stimulation. Remarkably, inducible nitric oxide synthase, IL-1β, TNF-α, reactive oxygen species and nitric oxide were significantly upregulated in activated BV-2 cells with CNPase knockdown. siRNA knockdown of CNPase increased microglia migration; on the other hand, microglial cells appeared to be arrested at G1 phase.

**Conclusions:**

The present results have provided the first morphological and molecular evidence that CNPase expression is increased in activated microglia. CNPase knockdown resulted in increased expression of various inflammatory mediators. It is concluded that CNPase may play an important role as a putative anti-inflammatory gene both in normal and injured brain.

**Electronic supplementary material:**

The online version of this article (doi:10.1186/s12974-014-0148-9) contains supplementary material, which is available to authorized users.

## Background

2′,3′-cyclic nucleotide 3′-phosphodiesterase (CNPase) is a protein that hydrolyzes cyclic nucleotides to monophosphates (2′ nucleotides). CNPase has two isoforms, CNPase1 (46 kDa) and CNPase2 (48 kDa) [1], which are encoded separately by different promoters of the same gene [2,3] and have different structural properties and subcellular localizations [4,5]. The majority of studies involving CNPase expression have focused on its roles in myelin-producing glial cells (oligodendrocytes and Schwann cells) and myelinogenesis. However, growing evidence has revealed the existence of this enzyme in non-myelinating cells, such as adrenal cells [6] and neuroblastoma cells (B104) [1]. Indeed, a considerable level of CNPase expression and CNPase-like enzyme activity were found in membranes of cells in the spleen, liver, kidney, heart, and skeletal muscles [7,8]. We have reported previously that CNPase expression was detected in bone marrow stromal cells and amoeboid microglial cells in the developing rat brain [9,10]. Interestingly, CNPase expression and its enzyme activity was localized in mitochondria, specifically in the mitochondrial membranes [6,11-13]. In the mitochondria isolated from CNPase knockdown OLN93 oligodendrocytes, a decrease in the CNPase level and its activity correlated with the acceleration of calcium release from the mitochondria [14]. These results demonstrate that CNPase may be implicated in the regulation of mitochondrial Ca^2+^ fluxes and may regulate the mitochondrial permeability transition pore. It is well documented that cellular Ca^2+^ signals are crucial in the control of most physiological processes. Mitochondria are considered as the main source of reactive oxygen species (ROS), which could cause cell injury, and they are the main site of the initiation of programmed cell death [15].

Previous localization of CNPase in amoeboid microglia had prompted us to further investigate the role of this enzyme in microglia, especially on activation. Microglial cells have been demonstrated to be activated by a variety of factors or stimuli and implicated in brain diseases and pathological processes, such as infections, trauma and inflammation [16,17]. Furthermore, microglial activation and its sequelae has been shown to be involved in neurodegenerative diseases and autoimmune diseases such as Parkinson’s disease, Alzheimer’s disease, and multiple sclerosis [17-19]. Microglia, when chronically activated, have been reported to cause neuronal damage through the release of potentially cytotoxic molecules such as proinflammatory cytokines, nitric oxide (NO) and ROS [20,21]. These mediators are thought to play contributory roles to inflammation-mediated neurodegenerative diseases [22]. Because CNPase expression coexists with the inflammatory mediators in microglia, which are enhanced on activation, it was surmised that they might regulate each other to perform the primary functions of microglia.

In light of the above, this study sought to determine if CNPase is expressed in activated microglia both *in vivo* and *in vitro*, and to elucidate how it might be regulated during microglial activation. To this end, upregulation of CNPase in microglia was elicited with lipopolysaccharide (LPS) treatment and downregulation of CNPase by its specific siRNA; this might help to unravel the specific roles of CNPase in microglia in normal and pathological conditions.

## Methods

### Animals

Wistar rats (3 days of age) were purchased from the Laboratory Animal Centre, National University of Singapore. All experiments were carried out in accordance with the International Guiding Principles for Animal Research, and approved by the Institutional Animal Care and Use Committee, National University of Singapore (protocol number NUS/ IACUC/2013-05512), and DSO Institutional Animal Care and Use Committee (protocol number DSO/IACUC/10/94). All efforts were made to minimize pain and the number of rats used.

### Injection of lipopolysaccharide

To investigate CNPase expression in activated microglia *in vivo,* 3-day-old postnatal rats were weighed and given a single intraperitoneal injection of LPS (1 mg/kg of body weight, diluted in 250 μl saline; Sigma-Aldrich, St Louis, MO, USA; Cat. No. L2654). In the control group (n = 3), LPS injections were replaced by an injection of saline (250 μl each pup). The pups were sacrificed at 1, 3, and 6 hours after LPS injection (n = 3 rats at each time point interval for each group). Frozen sections were prepared for immunohistochemistry.

### Fluid percussion injury

The lateral fluid percussion model of traumatic brain injury is one of the most extensively utilized animal models of traumatic brain injury due to its validity, reliability, clinical relevance and its usefulness in identifying cellular and molecular changes. The device consists of a Plexiglas cylindrical reservoir filled with distilled water or saline (Custom Design and Fabrication, Richmond, VA, USA). Sprague Dawley rats weighing between 250 and 280 g (n = 6 per group) were anesthetized with inhalational anesthesia (3 to 5% of isoflurane in oxygen at a flow rate of 1 l/minute) prior to the surgical procedure. The scalp was shaved and swabbed alternately with a chlorhexidane-alcohol swab. Thermoregulation was maintained by placing a warming pad (CMA 150, CMA Microdialysis, Kista, Sweden; or Right Temp Homeothermic warming system, Kent Scientific, Torrington, CT, USA) at 37 ± 0.5°C under the animal. With the aid of a stereotaxic atlas, a 4 mm hole was made at −3.8 mm bregma and left lateral 2.0 mm. A modified female Luer-Lok was twisted rigidly over the hole until the cannula abutted the dural surface. The pendulum was placed at a height that would inflict severe injury (~50 to 70 psi) by defining the force of the fluid pressure pulse transmitted through the saline reservoir. The burr hole was filled with collagen (Lyostypt®, B Braun Melsungen AG, Melsungen, Germany) and the incision sutured closed after the injury. Sham-operated animals received anesthesia and surgery, including probe removal and reinsertion but were not subjected to trauma treatment. The animals were sacrificed at 7 days post-injury and the brains were paraffin-fixed and prepared for immunohistochemistry.

### Stroke rat brain model

Sprague–Dawley rats weighing between 250 and 280 g were used. The rats were anaesthetized by an intraperitoneal injection of pentobarbital sodium (Ceva Sante Animale, Libourne, France; 50 mg/kg), and were fixed in the left lateral position. Following anesthesia, the rats were subjected to middle cerebral artery occlusion (MCAO). The surgical procedure followed that described previously by Wu and Ling [23]. Briefly, following incision of the skin, the right temporal muscle was excised and cleared until the underlying zygomatic arch was exposed. A circular hole, 3 mm in diameter, was burred in the right parietal bone with a dental drill and cold saline drip. The circular opening was enlarged with a rongeur by removing additional bone at the periphery to expose the main trunk of the middle cerebral artery (MCA). The pieces of bone removed were kept in cold saline. Care was taken not to damage the underlying cerebral cortex during craniotomy. The MCA was cauterized using a small vessel cauterizer (Fine Science Tools, North Vancouver, British Columbia, Canada), after which the bone flaps were replaced and the muscle and skin sutured separately, layer to layer. The rectal temperature was monitored and maintained between 37.5 and 38.5°C during the operation. On recovering from anesthesia, the rats showed signs of paresis of both the left limbs, especially the hind limb. In sham-operated rats, the same surgical procedure was carried out but the MCA was not cauterized. Along with MCAO and sham operated rats, normal rats (n = 9) of equivalent body weight were also used as controls. Brain tissues from rats subjected to MCAO (n = 9) were fixed with 4% paraformaldehyde. These tissues were paraffin-embedded and coronal sections at 7 μm thickness were cut on a microtome and collected on gelatin-coated slides for immunofluorescence staining.

### Double immunofluorescence staining *in vivo*

Normal postnatal control rats (n = 3) and LPS injected rats (n = 3, each at 1, 3 and 6 hours post-LPS injection) were used for immunofluorescence studies to detect the expression of CNPase in microglial cells. The rats were anesthetized with ketamine-xylazine cocktail (75 mg/kg and 10 mg/kg, respectively) and perfused with Ringer’s solution followed by 2% paraformaldehyde in 0.1 M phosphate buffer, pH 7.4. Following perfusion, the brains were removed, post-fixed for 4 hours in the same fixative and then cryoprotected in 15% sucrose at 4°C overnight. Frozen sections (30 μm thick) were cut coronally through the forebrain with a cryostat (Model CM 3050; Leica Instruments GmbH, Nubloch, Germany) and mounted onto gelatin-coated slides and stored at – 20°C until use. The brain sections at different timepoints were rinsed with PBS, blocked with 5% normal goat serum diluted in PBS for 1 hour at room temperature. Following removal of serum, tissue sections were incubated with mouse anti-human CNPase monoclonal primary antibody (dilution 1:100; Chemicon, Temecula, CA, USA; Cat. No. MAB 326) overnight at room temperature. On the next day, the sections were washed in PBS and incubated with a mixture of secondary antibodies: FITC-conjugated lectin from tomato (*Lycopersicon esculentum*) (1:200, Sigma-Aldrich; Cat. No. L-0401) and Cy3-conjugated goat anti-mouse IgG (1:200, Sigma-Aldrich; Cat. No. T5393) for 1 hour at room temperature. The sections were then washed in PBS and mounted using a fluorescent mounting medium (Dako, Oregon City, USA; Cat. No. S3023). Cellular localization was then examined under a confocal microscope (Fluoview 1000; Olympus, Tokyo, Japan) with the same exposure settings for each comparison group. Tissue sections derived from MCAO and fluid perfusion injury (FPI) were processed with the immunofluorescence staining methods as described above.

### Primary culture and lipopolysaccharide treatment of microglial cells

Three-day-old postnatal rats were also used for the preparation of primary culture of microglia (n = 36). Increased CNPase expression was observed in activated microglia in preliminary immunofluorescence labeling. In view of this, primary culture of microglia was prepared for *in vitro* investigations. Glial cells were isolated from the brains of rat pups and were placed in a 75 cm^2^ flask at a density of ~1.2 × 10^6^ cells/ml of DMEM (Sigma-Aldrich) supplemented with 20% fetal bovine serum (FBS; Hyclone, Thermo Scientific, Waltham, MA, USA) and 1% antibiotic antimycotic solution (Sigma-Aldrich; Cat. No. A5955). The flasks were then placed in a humidified atmosphere containing 5% CO_2_ and 95% air at 37°C. The culture medium was changed every 48 to 72 hours. Microglia were isolated from the mixed glial population by a method described previously [24] when mixed glial cells were confluent (12 to 14 days). The purity of microglia was assessed by immunofluorescence labeling using FITC-conjugated lectin (a marker of microglia) from tomato (*Lycopersicon esculentum*) (1:200, Sigma-Aldrich; Cat. No. L-0401). Microglial cultures with more than 96% purity were used for the study. For immunostaining, ~2.5 × 10^5^ cells/well were plated on poly-L-lysine coated coverslips placed in 24-well plates. For LPS treatment, primary cultured microglial cells were stimulated with 0.5 μg/ml LPS in DMEM supplemented with 10% FBS for 24 hours.

### BV-2 cell culture

Murine BV-2 microglial cell lines were maintained in 75 cm^2^ culture flasks in DMEM supplemented with 2% FBS and 1% antibiotic antimycotic solution at 37°C in a humidified atmosphere of 5% CO_2_ and 95% air. Cells were plated on 24-well plates at a density of ~6.0 × 10^4^ per well for immunocytochemistry and at ~3 × 10^5^ per well on a 6-well plate for gene silencing CNPase transfection studies, RNA isolation, protein extraction and flow cytometric assay. On the following day after plating, cells were subjected to different treatments. The time point (6 hours) and dosage (1.0 μg/ml) of LPS (Sigma-Aldrich; Cat. NO. L2654) treatment were chosen based on results obtained from carrying out the MTS assay from previous reports [25].

### Immunofluorescence staining of microglial cells *in vitro*

Primary microglial cells and BV-2 cells were plated on poly-L-lysine coated cover slips in a 24-well plate. For immunofluorescence labeling, the cells subjected to different treatments of varying durations were fixed with 4% paraformaldehyde for 20 minutes at room temperature and blocked with 5% normal goat serum for 1 hour. The cells were then separately incubated overnight at 4°C with mouse anti-human CNPase monoclonal antibody (1:100; Chemicon; Cat. No. MAB326), rabbit anti-mouse TNF-α polyclonal antibody (1:100, Millipore Bioscience Research Reagents, Billerica, MA, USA; Cat. No. AB2148P), rabbit anti-mouse IL-1β polyclonal antibody (1:100, Millipore Bioscience Research Reagents; Cat. No. AB1413), and mouse anti-mouse inducible nitric oxide synthase (iNOS) monoclonal antibody (1:100, BD Pharmingen, San Jose, CA USA; Cat. No.640432), followed by Cy3-conjugated sheep anti-mouse IgG (1:200, Sigma-Aldrich; Cat. No. C2181) or Cy3-conjugated sheep anti-rabbit IgG secondary antibodies (1:200, Sigma-Aldrich; Cat. No. C2306) incubation for 1 hour at room temperature. The cells were then washed three times with PBS and mounted using a fluorescent mounting medium (Sigma-Aldrich; Cat. No. F6057). Cellular localization was then examined under a confocal microscope (Fluoview 1000; Olympus) with the same exposure settings for each comparison group.

### Silencing of CNPase with siRNA

Two constructs of mouse CNPase-specific siRNA (Ambion, Foster City, CA, USA; siRNA ID: s64160 & s64161; Cat. No. 4390771) were used for CNPase silencing. Nonspecific scramble siRNA (Ambion; Cat. No. 4390846) was used as control siRNA. To achieve a higher siRNA knockdown efficiency, the reverse transfection method was adopted for silencing according to the manufacturer’s instructions. Briefly, sub-confluent, early passage BV-2 cells were harvested by trypsinization, centrifuged and resuspended in Optimem (GIBCO, Invitrogen, Carlsbad, CA, USA; Cat. No. 31985070) and plated in 6-well plates at a density of ~3 × 10^5^ cells/well. This was followed by adding 500 μl Optimem with 5 μl siRNA (10 μM) and 4 μl Lipofectamine® RNAiMAX Transfection Reagent (Invitrogen; Cat. No. 13778075) dropwise in the above well. The cells were incubated with the siRNA transfection mixture for 8 hours and then the medium was replaced with DMEM with 10% FBS without antibiotics and incubated for another 16 hours for RNA extraction to detect the knockdown efficiency by reverse transcription (RT)-PCR. Microglial cells were subjected to LPS treatment for 6 hours at 42 hours after transfection. After that, cells were either fixed for immunofluorescence staining, or mRNA and protein extracted for real-time RT-PCR and western blotting, respectively.

### Cell viability analysis of BV-2 cells

The effect of siRNA transfection on the viability of BV-2 cells was evaluated by CellTiter 96® AQueous One Solution Cell Proliferation Assay kit (Promega, Fitchburg, WI, USA; Cat. No. G3580). The cell viability of non-transfected BV-2 cells, control siRNA transfected BV-2 cells, and CNPase siRNA transfected BV-2 cells were measured. The dye solution was added to the cells at 24 hours post-transfection and incubated for up to 4 hours at 37°C in a humidified, 5% CO_2_ incubator. The absorbance was read at 490 nm using the Tecan 2000 microplate reader. Cell viability was expressed as a percentage of non-transfected BV-2 cells.

### Cell cycle analysis

For each experiment, control siRNA and CNPase siRNA transfected cells were harvested at 48 hours post-transfection and pelleted by centrifugation. The cell pellet was resuspended in 0.5 ml PBS and fixed in 4.5 ml 70% cold ethanol overnight at 4°C. Ethanol-fixed cells were washed twice with PBS before resuspending with 200 μg/ml propidium iodide solution containing 1 mg/ml RNAse A, PBS and Triton-X. Cells were incubated at room temperature for 30 minutes to allow the DNA content of cells to be stained before analysis by flow cytometry.

### Real-time reverse-transcription PCR

The total RNA was extracted from primary microglial cells and BV-2 microglial cells subjected to various treatments (LPS, control and CNPase siRNA transfection) using RNeasy Mini kit (Qiagen, Valencia, CA, USA). The concentration of RNA was quantified with Nanodrop Spectrophotometer (Thermo Scientific; Model No. ND1000). RNA (1 μg) was reverse transcribed to cDNA using the SuperScript® III First-Strand Synthesis System (Invitrogen; Cat. No 18080–051) according to the manufacturer’s protocol. The resulting cDNA was diluted and used as a template for real-time reverse transcription PCR using an ABi 7900HT Fast PCR system (Applied Biosystems, Grand Island, NY, USA) according to the manufacturer’s instructions. Primer pairs for *CNPase, TNF-α, IL-1β, iNOS* and *β-actin* were designed using the primer design program (Primer 3 software version 1.0, Whitehead Institute for Biomedical Research, Cambridge, MA, USA). The primer sequences for the genes are listed in Table 1. The RT-PCR was carried out in a 10 μl final volume containing the following: 5 μl 2 × SYBR Green fast master mix (Invitrogen); 1 μl 5 μM forward primer and 1 μl 5 μM reverse primer; and 3 μl diluted cDNA. After an initial denaturation step at 95°C for 15 minutes, temperature cycling was initiated. Each cycle consisted of denaturation at 94°C for 15 seconds, annealing at 60°C for 25 seconds, and elongation at 72°C for 20 seconds. In total, 45 cycles were performed. Mouse β-actin was amplified as the control for normalizing the quantities of transcripts of each of the genes mentioned above. The differences in expression for *CNPase*, *TNF*-*α*, *IL-1β* and *iNOS* between the control and treated cells were calculated by normalizing with the *β-actin* gene expression according to the following formula [26]:

**Table 1:** **Sequence of specific primers used for quantitative real-time reverse transcription PCR**

**Gene Sequence**		
CNPase	Forward	gcaggaggtggtgaagagat
	Reverse	cagatggcttgtccagatca
TNF-α	Forward	cgtcagccgatttgctatct
	Reverse	cggactccgcaaagtctaag
IL-1β	Forward	gcccatcctctgtgactcat
	Reverse	aggccacaggtattttgtcg
iNOS	Forward	gcttgtctctgggtcctctg
	Reverse	ctcactgggacagcacagaa
β-actin	Forward	ggattccatacccaagaagga
	Reverse	gaagagctatgagctgcctga

CNPase, 2′,3′-cyclic nucleotide 3′-phosphodiesterase; IL-1β: interleukin-1beta; iNOS, inducible nitric oxide synthase; PCR, polymerase chain reaction; TNF-α, tumor necrosis factor alpha.

$$ \mathrm{Fold}\ \mathrm{change} = {2}^{-\left[ Ct(control) gene\times - Ct(control) actin\right]-\left[ Ct(activated) gene\times - Ct(activated) actin\right]} $$

### Western blotting analysis

Cells were washed twice with cold PBS and lysed using M-PER® Mammalian Protein Extraction Reagent (Thermo Fish Scientific Inc., Rockford, IL, USA; Cat. No. 78501) containing protease inhibitor cocktail (Thermo Fish Scientific Inc.; Cat. NO. 78410) and nuclear protein extraction kit (Millipore; Cat. No. 2900). The lysate was collected by centrifugation at 13,000 rpm for 20 minutes at 4°C. The amount of protein was quantified using a protein assay kit (Bio-Rad, Hercules, CA, USA; Cat. No. 500–0002) and 20 μg of protein sample was loaded to 10% sodium dodecyl sulfate-polyacrylamide gel and separated by electrophoresis. Separated proteins were then transferred to polyvinylidene difluoride membranes using a semidry electrophoretic transfer cell (Bio-Rad). The membranes were blocked with 5% non-fat milk for 1 hour at room temperature, then they were incubated with mouse anti-human CNPase monoclonal antibody (1:1000, Chemicon), rabbit anti-mouse TNF-α polyclonal antibody (1:1000, Millipore), rabbit anti-mouse IL-1β polyclonal antibody (1:1000, Millipore), mouse anti-mouse iNOS monoclonal antibody (1:500, BD Pharmingen), rabbit anti-human NF-κB monoclonal antibody (1:1000; Cell Signaling, Beverley, MA, USA; Cat. No. 4764), mouse anti-human β-actin monoclonal antibody (1:10,000; Sigma-Aldrich; Cat. No. A5441) and rabbit anti-mouse Lamin A polyclonal antibody (1:1000; Santa Cruz, Dallas, TX, USA; Cat. No. sc-20680) overnight on a shaker at 4°C. After three washes with TBS-0.1% Tween, the membranes were incubated with horseradish peroxidase-conjugated secondary antibody for 1 hour. The proteins were detected with a chemiluminescence detection system according to the manufacturer’s instruction (Supersignal West Pico Horseradish Peroxidase Detection Kit; Pierce Biotechnology, Rockford, IL, USA; Cat. No. 34077) and developed on the film. The band intensity was quantified in Image J software (National Institutes of Health, USA). Each lane of protein band density was normalized with β-actin.

Measurement of reactive oxygen species production in BV-2 microglia by flow cytometry

Briefly, BV-2 cells were grown in a 6-well plate at a density of ~3.0 × 10^5^ per well and treated as described above. After that, cells were washed twice with PBS. Staining was performed for 40 minutes at 37°C in the dark with 10 μmol/L CM-H2DCFDA (Molecular Probers, Grand Island, NY, USA; Cat. No. C6827). Cells were washed twice with PBS and harvested by trypsinization, centrifuged and resuspended in PBS, then immediately measured with a flow cytometer (BD Biosciences, San Jose, CA, USA). The data obtained were analyzed with FlowJo software, Flowjo LLC, Ashland, OR, USA).

### Nitric oxide concentration measurement

Cell culture medium was collected from control siRNA or CNPase siRNA transfected BV-2 cells with and without LPS treatment. The NO concentration in the culture medium was quantified by a Griess reagent system NO colorimetric BioAssay™ Kit (US Biological, Swampscott, MA, USA, Cat. No N2577-01) according to the manufacturer’s instruction. The absorbance at 540 nm was determined with a microplate reader (GENIOS, Tecan, Switzerland).

### Statistical analyses

The GraphPad Prism software (San Diego, CA, USA) was used for statistical analysis. The data were expressed as mean ± SD. Unpaired *t*-test and one-way analysis of variance were performed for comparison of two and more than two groups in the *in vitro* experiments, respectively. *P < 0.05, **P < 0.01 and ***P < 0.001 were considered as statistically significant.

## Results

### CNPase expression is increased in microglia after lipopolysaccharide treatment in postnatal rats

**Figure 1:**
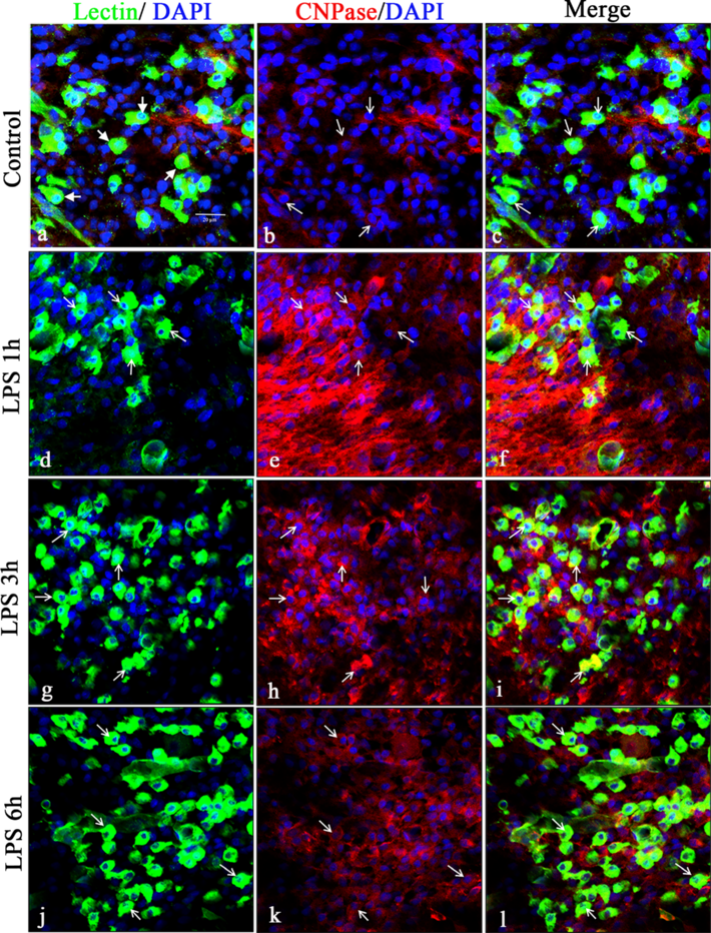
**CNPase immunofluorescence was increased in the brain in postnatal rats following lipopolysaccharide injection.** Confocal images showing the distribution of lectin (green), CNPase (red) and DAPI (blue) immunoreactive cells in the postnatal rat brain at 1 **(d-f)**, 3 **(g-i)** and 6 hours **(j-l)** after lipopolysaccharide (LPS) treatment and the corresponding control **(a-c)**. Colocalized expression of CNPase in lectin immunoreactive cells (arrows in c, f, i, l) can be seen. Note the upregulated expression of CNPase in some lectin-positive microglial cells after LPS treatment (f, i, l,). Arrows indicate microglial cells expressing both lectin and CNPase. Scale bar= = 20 μm **(A)**. CNPase, 2′,3′-cyclic nucleotide 3′-phosphodiesterase; DAPI, 4′,6- diamidino-2-phenylindole.

**Figure 2:**
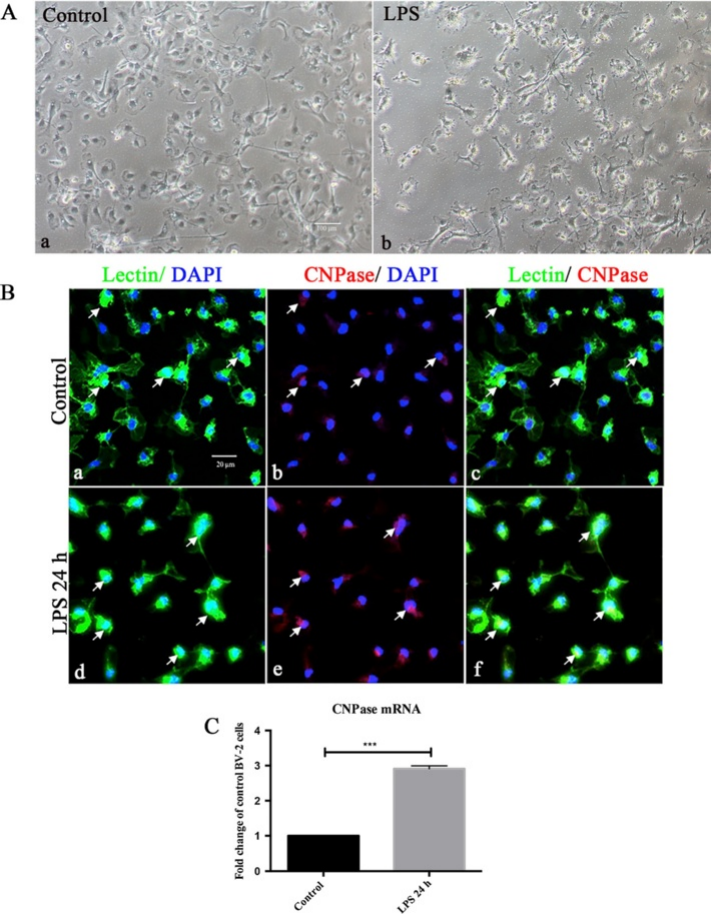
**CNPase expression was increased in primary cultured microglia following lipopolysaccharide treatment. (A)** Note the change in external morphology of primary microglial cells bearing long extending and stout processes after lipopolysaccharide (LPS) treatment (Ab) in comparison with the control (Aa) under the phase contrast microscope. **(B)** Confocal images showing CNPase expression (Bb, Be; red) in primary microglia labeled with lectin (Ba, Bd; green) and DAPI (blue) in both control and LPS treatment for 24 hours. CNPase immunofluorescence intensity is enhanced after LPS treatment (Bf) in comparison with the control (Bc). **(C)** CNPase mRNA expression in control and LPS activated primary microglia. LPS stimulated primary microglial cells show a significant upregulation of CNPase mRNA in comparison with the control cells. ***P < 0.001. The values represent the mean ± SD in triplicate. Scale bars = 100 μm **(A)** and 20 μm **(B)**. CNPase, 2′,3′-cyclic nucleotide 3′-phosphodiesterase; DAPI, 4′,6- diamidino-2-phenylindole.

To investigate the function of CNPase in microglial cells in the developing brain with LPS stimulation, we profiled the expression of CNPase in activated microglia in the brain, as well as primary cultured microglial cells subjected to LPS treatment. *In vivo*, CNPase expression was colocalized in lectin-labeled amoeboidic microglial cells in the brain (Figure 1). At 1, 3 and 6 hours after LPS treatment, CNPase expression in sporadic microglia was markedly increased (Figure 1). Consistent with results *in vivo*, mRNA expression and immunoexpression changes in CNPase were also upregulated in the primary cultured microglia. CNPase immunofluorescence intensity was enhanced (Figure 2B) and mRNA expression (Figure 2C) was significantly increased more than three-fold versus control when primary microglial cells were subjected to LPS treatment for 24 hours. It is noteworthy that microglial cells exhibited many long extending and stout processes after 24 hours of LPS treatment (Figure 2A).

### CNPase expression is increased in microglia after middle cerebral artery occlusion and fluid perfusion injury in adult rats

**Figure 3:**
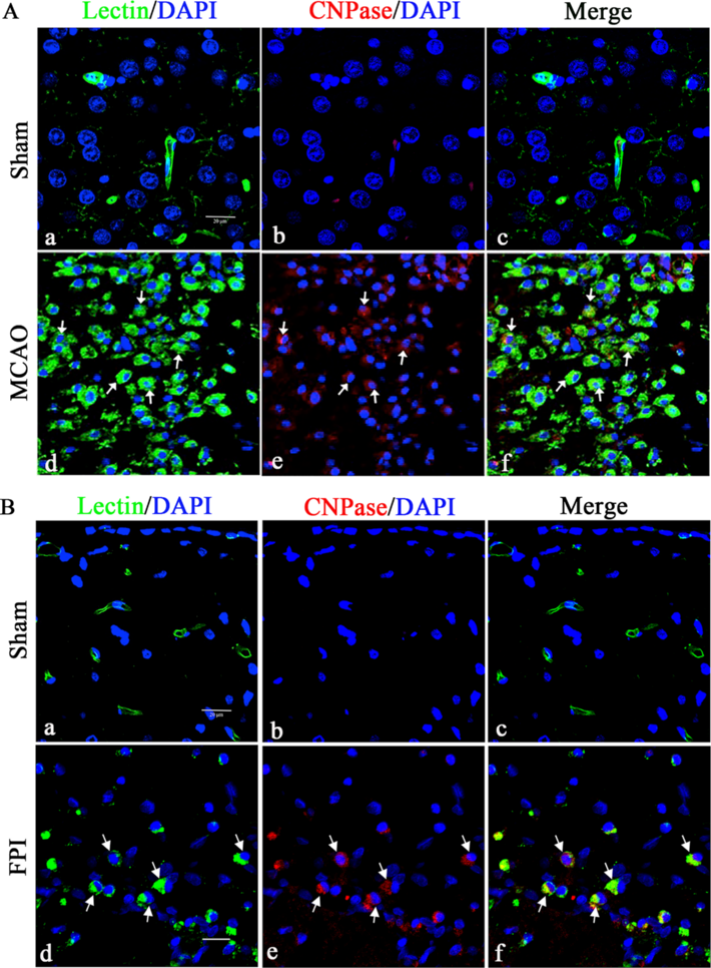
**Confocal images showing the induction of CNPase expression at 7 days after middle cerebral artery occlusion and fluid perfusion injury.** CNPase expression (Ab, Ae, and Bb, Be; red) in FITC-lectin labeled microglia (Aa, Ad, and Ba, Bd; green,) and DAPI (blue) in the ischemic cortex **(A)** and ipsilateral injury cerebrum **(B)** is hardly detected in the microglia in the sham group. A marked increase in CNPase expression is observed in FITC-lectin-labeled microglia both in the middle cerebral artery occlusion (MCAO) infarct area and fluid perfusion injury (FPI) site when compared to the sham group. Note also that the activated microglial cells are amoeboidic in appearance. Scale bars = 20 μm (A and B). CNPase, 2′,3′-cyclic nucleotide 3′-phosphodiesterase; DAPI, 4′,6- diamidino-2-phenylindole.

Since microglia is activated in response to brain injury, we have analyzed CNPase expression in activated microglia in the cerebrum from MCAO and FPI rat models. Enhanced CNPase expression was localized in activated microglial cells in the ischemic cortex of the cerebral hemisphere (Figure 3A) as well as in the FPI injury cortex (Figure 3B) when compared with the sham group.

### Silencing of CNPase gene in BV-2 cells

**Figure 4:**
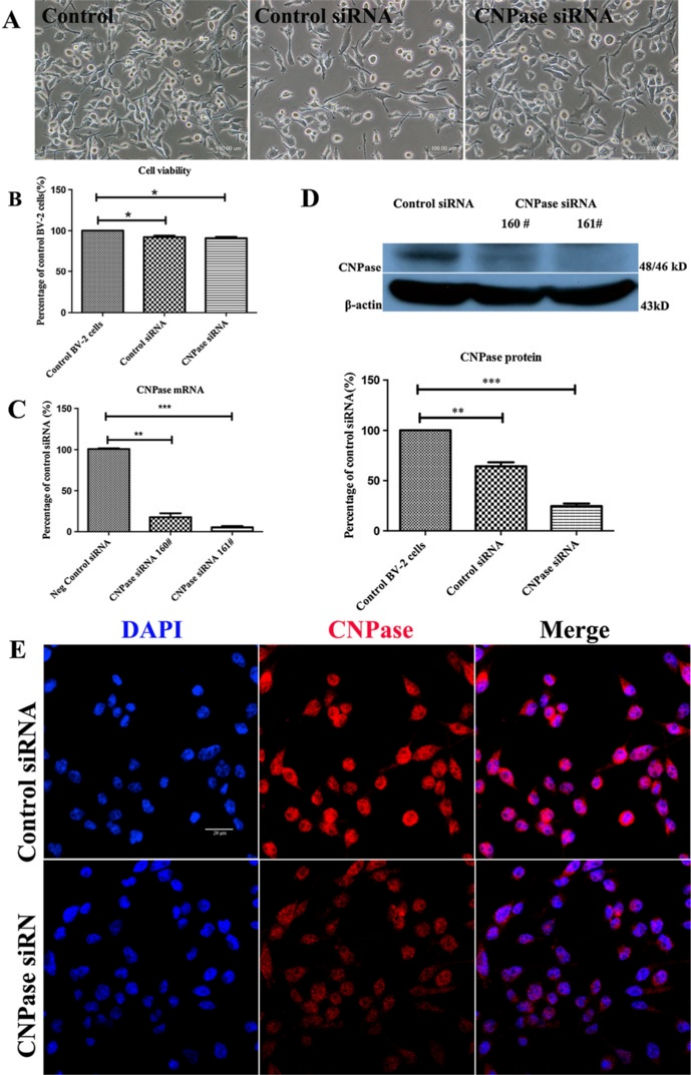
**Downregulation of CNPase after CNPase siRNA transfection in BV-2 cells. (A)** There was no noticeable change in external morphology in BV-2 cells when transfected with either control small interfering RNA (siRNA) or CNPase siRNA, and when compared with the non-transfected control cells under the phase-contrast microscope. **(B)** The viability of BV-2 cells transfected with control siRNA and CNPase siRNA is 92% and 91%, respectively, against the non-transfected control value. **(C)** Reverse transcription polymerase chain reaction analysis shows that the efficiency of siRNA (160 #)-mediated suppression of CNPase is about 84% while that of siRNA (161 #) is about 94% compared to negative control (normalized with β-actin). **(D)** The upper panel shows the specific Western band of CNPase and β-actin proteins. The lower panel shows bar graphs depicting significant changes in the optical density of different groups. Note the remaining CNPase protein expression in CNPase siRNA (161 #) transfected BV-2 cells is about 25% compared to the control siRNA transfected BV-2 cells. **(E)** Immunofluorescence images show CNPase immunoreactivity is markedly reduced in CNPase siRNA transfected BV-2 cells compared to negative control. *P < 0.05, **P < 0.01 and ***P < 0.001. The values represent the mean ± SD in triplicate. Scale bars = 100 μm **(A)** and 20 μm **(E).** CNPase, 2′,3′-cyclic nucleotide 3′-phosphodiesterase; DAPI, 4′,6- diamidino-2-phenylindole.

To further determine roles of CNPase in microglial cells after LPS treatment, siRNA was employed to knockdown the *CNPase* gene in BV-2 cells. BV-2 cells were either transfected with control siRNA or CNPase siRNA. The external morphology of transfected BV-2 cells was comparable to the non-transfected BV-2 cells under the phase-contrast microscope (Figure 4A). Compared with the non-transfected control BV-2 cells, the cell viability was about 92% and 91% after control siRNA and CNPase siRNA transfection, respectively (Figure 4B). Quantitative RT-PCR was carried out using cDNA obtained from BV-2 microglia treated with two different siRNA constructs (160# and 161#) against *CNPase* mRNA. It was observed that construct 161# produced the maximum CNPase knockdown efficiency, which was achieved at about 94% at 24 hours post-transfection when compared to control siRNA transfected cells (Figure 4C), and hence was used for subsequent functional analysis. Moreover, downregulation of the CNPase protein was verified by western blot analysis at 48 hours post-transfection which showed a reduction in the expression of CNPase protein by 75% (Figure 4D). Confocal immunofluorescence microscopy also showed an obvious reduction in CNPase immunostaining intensity in CNPase knockdown BV-2 cells (Figure 4E).

### Knockdown of CNPase expression in BV-2 cells prompted increased production of inflammatory mediators

**Figure 5:**
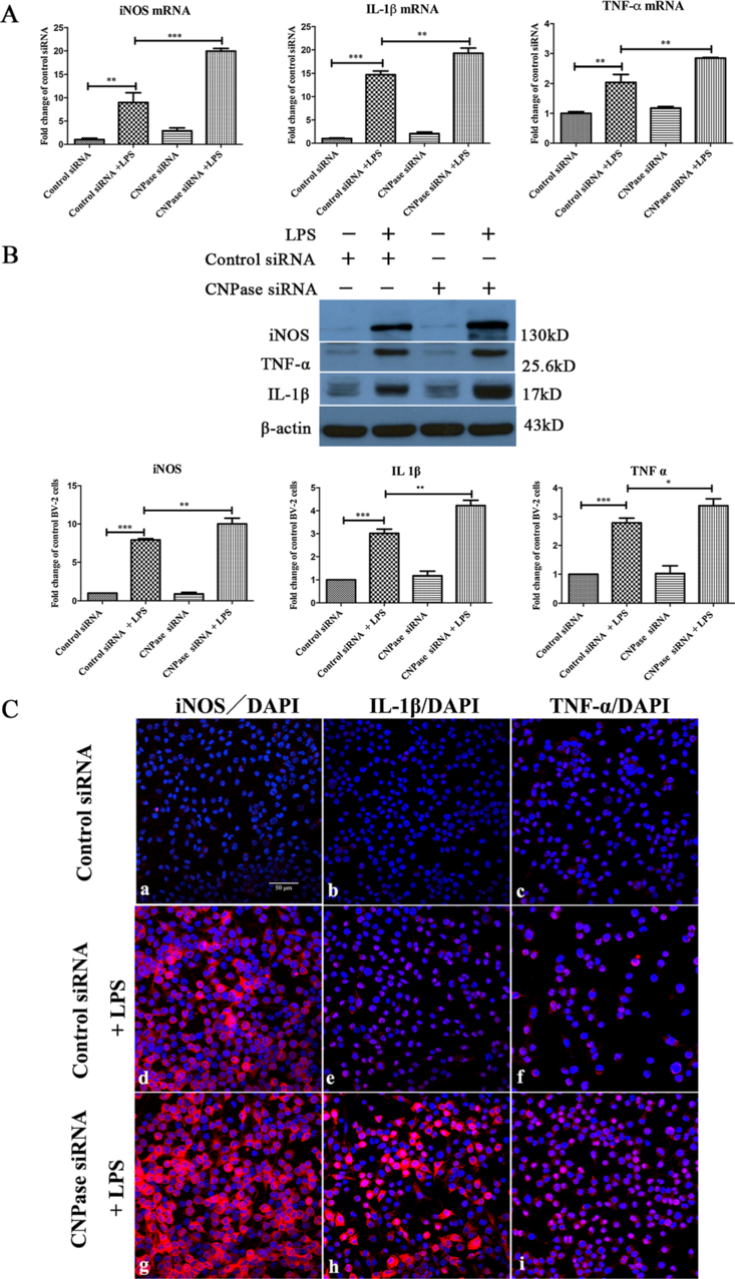
**CNPase knockdown increased inducible nitric oxide synthesis, IL-1**β **and TNF-**α **expression induced by lipopolysaccharide in BV-2 cells. (A)** Reverse transcription polymerase chain reaction analysis of *iNOS*, *IL-1β* and *TNF-α* gene expression in BV-2 cells transfected with control small interfering RNA (siRNA), transfected with control siRNA + lipopolysaccharide (LPS), and transfected with CNPase siRNA and CNPase siRNA + LPS. Note that *iNOS*, *IL-1β* and *TNF-α* mRNA expression is increased by different amounts, respectively, after LPS treatment in control siRNA transfected BV-2 (normalized with β-actin), but the increase was significantly higher in LPS stimulated CNPase siRNA transfected BV-2 cells compared with control siRNA transfected BV-2 cells exposed to LPS stimulation. **(B)** iNOS, IL-1β and TNF-α protein expression in different groups of BV-2 cells given LPS treatments. The upper panel shows specific bands of iNOS, IL-1β ,TNF-α and β-actin. The lower panel of the bar graphs shows significant changes in the optical density following LPS treatment (given as fold-change of control siRNA transfected group). iNOS, IL-1β and TNF-α protein expression is significantly increased after LPS treatment in control siRNA transfected BV-2 cells and the increase is further augmented after LPS stimulation in CNPase transfected groups. **(C)** Immunofluorescence images show iNOS, IL-1β and TNF-α immunoreactivity is markedly increased in CNPase siRNA transfected BV-2 cells subjected to LPS treatment compared to cells transfected with control siRNA followed by LPS treatment. *P < 0.05 and **P < 0.01. The values represent the mean ± SD in triplicate. Scale bar = 50 μm **(C)** . CNPase, 2′,3′-cyclic nucleotide 3′-phosphodiesterase; IL-1β, interleukin-1 beta; iNOS, inducible nitric oxide synthase; TNF, tumor necrosis factor alpha.

**Figure 6:**
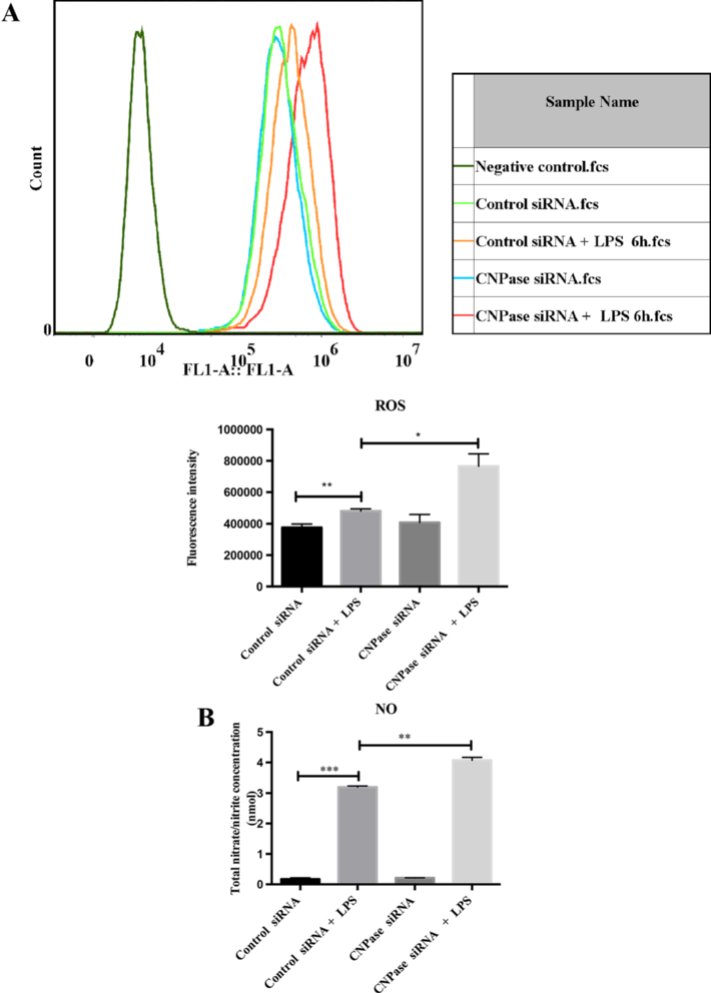
**CNPase knock down increased the production of reactive oxygen species and nitric oxide induced by lipopolysaccharide stimulation in BV-2 cells. (A)** Intracellular reactive oxygen species (ROS) and production of nitric oxide (NO) in BV-2 cells transfected with control small interfering RNA (siRNA), control siRNA + lipopolysaccharide (LPS) 6 hours, and transfected with CNPase siRNA and CNPase siRNA + LPS 6 hours. **(A)** The upper panel shows cell counts (y-axis) and log10 expression of fluorescence intensity (x-axis). The lower panel is a bar graph showing a significant change in the fluorescence intensity of intracellular ROS production following the various treatments. Note that the increase of ROS production in CNPase siRNA transfected BV-2 cells with LPS treatment is higher than that in control siRNA transfected BV-2 cells with LPS stimulation. **(B)** NO production in supernatant shows a similar change as with ROS in the different groups mentioned above. *P < 0.05, **P < 0.01 and ***P < 0.001. The values represent the mean ± SD in triplicate. CNPase, 2′,3′-cyclic nucleotide 3′-phosphodiesterase; fcs, fluorescence-activated cell sorting.

mRNA expression of LPS-induced inflammatory mediators including *iNOS, IL-1β* and *TNF-α* was drastically elevated in CNPase silencing BV-2 cells with LPS treatment. The most remarkable increase was observed in *iNOS* mRNA expression, which was increased nine-fold in control siRNA transfected BV-2 cells with LPS treatment for 6 hours. The increase was even more dramatic, nearing twenty-fold in CNPase silencing BV-2 cells with exposure to LPS for the same duration (Figure 5A). A similar trend was observed in *IL-1β* and *TNF-α* mRNA expression levels across the different groups of cells subjected to various treatments. In parallel with mRNA expression, western blot results showed that protein expression levels of iNOS, TNF-α, and IL-1β were significantly increased in LPS treated BV-2 cells with CNPase knockdown compared with those transfected with control siRNA (Figure 5B). As in mRNA expression level, iNOS protein expression level was highest when compared with that of TNF-α and IL-1β. This was further verified by confocal immunofluorescence microscopy, which showed an obvious increased expression in iNOS, IL-1β and TNF-α immunostaining intensity in LPS treated BV-2 cells with CNPase knockdown compared with the LPS-stimulated control siRNA transfected BV-2 cells (Figure 5C). Flow cytometry results revealed that intracellular ROS production was significantly increased in control siRNA transfected BV-2 cells with LPS treatment, and this increase was more significant in activated BV-2 cells with CNPase knockdown when compared with the former (Figure 6A). Moreover, similar changes in NO concentration in the supernatant derived from the different treatments mentioned above were observed (Figure 6B).

### Effects of CNPase knockdown on cell cycle progression

**Figure 7:**
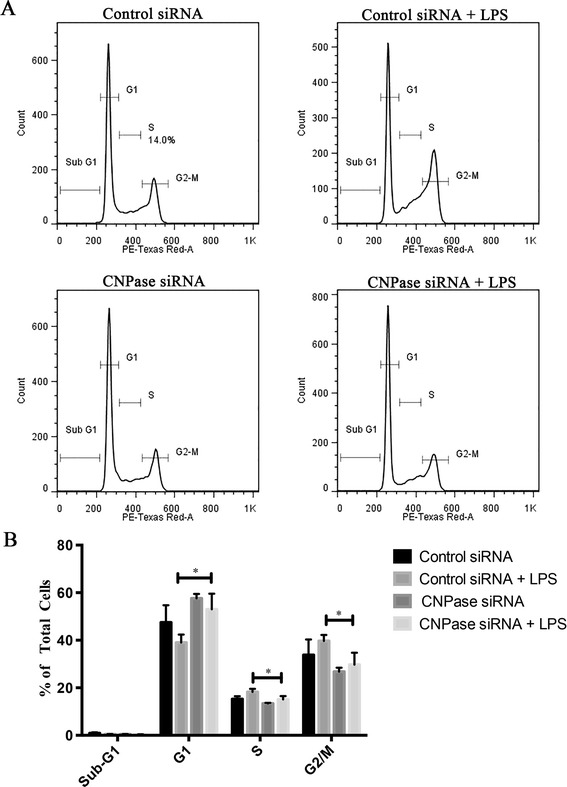
**CNPase knockdown on cell cycle progression in BV-2 cells. (A)** Representative DNA histogram plots from an individual experiment showing control small interfering RNA (siRNA) and CNPase siRNA transfected groups with and without lipopolysaccharide (LPS) treatment. **(B)** Percentage of total cells in each phase of cell cycle. CNPase knockdown arrested the cells in the G1 phase with a corresponding decrease in the percentage of cells entering into the S + G2/M phase. *P < 0.05 compared with controls by unpaired *t*-test, from three independent experiments. CNPase, 2′,3′-cyclic nucleotide 3′-phosphodiesterase.

Cell-cycle progression of BV-2 cells with or without CNPase knockdown was examined. As shown in Figure 7, in activated BV-2 cells, silencing of the CNPase gene increased the percentages of cells in G1 phases and decreased the percentages of cells in the S and G2/M phases (Figure 7). There was no significant change in the percentage of apoptotic cells in CNPase siRNA transfected cells as compared to control siRNA transfected cells. CNPase siRNA transfected cells showed a significantly higher percentage of cells in the G1 phase (53%) as compared to control siRNA treated cells (39%). A decrease in the number of cells in S phase was also observed for CNPase siRNA transfected cells as compared to control siRNA treated cells (15.1% compared with 18.2%). For the G2/M phase, there was a significant reduction in the percentage of cells in CNPase siRNA treated cells (39.7%) as compared with control siRNA treated cells (29.7%). The sum total of the S phase and G2/M phase, which is indicative of cell proliferation, was significantly higher in control siRNA treated cells (57.9%) as compared with CNPase siRNA treated cells (44.8%).

### Effects of CNPase knockdown on NF-κB transcriptional activity

**Figure 8:**
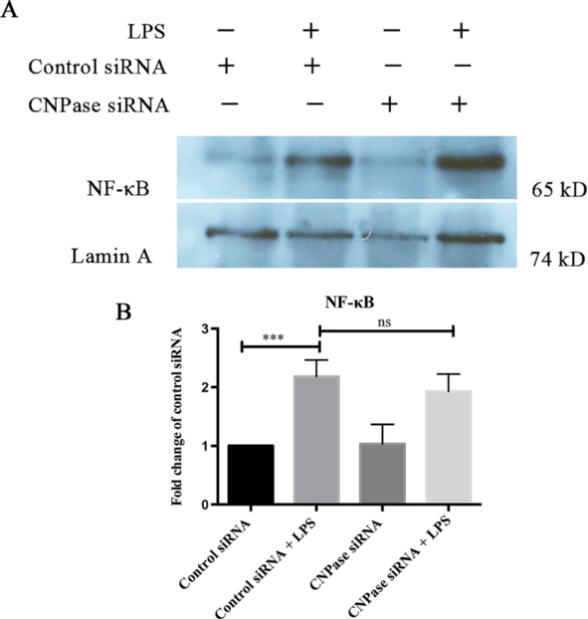
**CNPase knockdown does not affect the nuclear translocation of NF-**κ**B in activated BV-2 microglial cells. (A)** Western blot shows the expression of NF-κB (65 kDa) and Lamin A (74 kDa) in nuclear protein isolated from the various groups. **(B)** Expression of nuclear NF-κB protein was significantly increased in BV-2 cells exposed to lipopolysaccharide (LPS) for 3 hours, and this increase has no significance between control small interfering RNA (siRNA) and CNPase siRNA tranfected BV-2 cells. Data are presented as mean ± SD (n = 3), ***P < 0.001; ns, no significant difference. CNPase, 2′,3′-cyclic nucleotide 3′-phosphodiesterase.

NF-κB is a nuclear transcription factor that plays a pivotal role in immune and inflammatory responses by regulating genes encoding proinflammatory cytokines, adhesion molecules, chemokines, growth factors, and inducible enzymes. NF-κB was significantly increased in BV-2 cells with LPS treatment for 3 hours. However, this increase is comparable to activated BV-2 cells with CNPase knockdown (Figure 8).

## Discussion

CNPase is named after its ability to catalyze the phosphodiester hydrolysis of 2′,3′-cyclic nucleotides to 2′-nucleotides [27]. However, the physiologically relevant substrates and the specific biological roles of CNP in mammalian cells had remained to be fully clarified. CNPase is present in diverse eukaryotes and it is reported to be expressed at low levels in many mammalian tissues, at higher levels in dendritic cells, and at extremely high levels in oligodendrocytes, the major glial cells in the human brain [28-30]. CNPase is expressed as two isoforms, with CNPase 2 identical to CNPase 1 with a 20 amino acid extension at the N-terminus of CNPase 2 [31]. Mitochondrial CNPase localization was also observed in glial (C6) and non-glial cells (CHO, NIH 3 T3, HeLaS3) [12,32]. In our studies, it was observed that mitochondrial CNPase protein was significantly higher than that in cytosol in BV-2 microglial cells (Additional file 1).

We previously reported CNPase expression in the amoeboid microglial cells transiently occurring in the perinatal brain [9]. It was further shown that CNPase was markedly elevated in activated microglia. In view of this, it was suggested that CNPase might be linked to the major functions of the activated microglia such as release of chemokines and cytokines, but the specific functional roles of the enzyme in microglia remains uncertain. Here, we extended the study and demonstrated additional roles of microglial CNPase, such as its ability to inhibit the production of proinflammatory mediators, besides its well-documented maintenance function in oligodendrocytes.

CNPase was found to be induced in activated microglia, not only in the developing brain and primary microglial cultures (Figure 2), but also in activated microglia in traumatic brain injury and experimentally induced cerebral ischemia in adult rats (Figure 3). Whether the increased CNPase expression was caused by the inflammation-associated cytokines is still unknown. Notwithstanding, since increased CNPase expression was detected in activated microglia, a key player in neuroinflammation, it is proposed that the enzyme might be linked to inflammatory responses as discussed below.

Microglia play a pivotal role in the innate central nervous system immune response by facilitating neuroprotection and repair processes against invading pathogens [33]. During neuroinflammation, activated microglia lead to clearance of debris or invading pathogens, and release of neurotrophic factors that regulate the microenvironment [34,35]. However, in response to injury, microglia transform into a more motile state and migrate to the site of damage [36,37]. These activated microglia produce a variety of pro-inflammatory mediators, such as the small gas molecule NO, and pro-inflammatory cytokines, such as TNF-α and IL-1β. These molecules have been reported to play important roles in the initiation and progression of severe neurodegenerative diseases [38,39]. We showed here that CNPase is constitutively expressed in microglia and that it is augmented after LPS treatment. Based on this premise, we adopted siRNA transfection to knockdown CNPase mRNA expression to further elucidate its roles in activated microglia. Remarkably, siRNA CNPase knockdown affected the expression of pro-inflammatory cytokines. More specifically, lowering CNPase expression significantly promoted mRNA and protein expression of iNOS, IL-1β and TNF-α induced by LPS treatment, suggesting that CNPase may have a suppression effect on iNOS, IL-1β and TNF-α production in activated microglial cells.

Along with iNOS, IL-1β and TNF-α, NO and ROS productions were increased after siRNA CNPase knockdown with LPS treatment. The pro-inflammatory mediator NO is the product of the inducible isoform of NOS [40]. NO is a signaling molecule involved in a wide range of physiological and pathological processes. Examples of its involvement include neural transmission and immune response. An excess production of NO can also lead to the initiation and maintenance of inflammation [41,42]. In this study, lowering CNPase expression significantly promoted LPS-induced release of NO in BV-2 cells when compared with the control siRNA group. This suggests that CNPase may act to suppress production of NO and hence modulate the neuronal and immune response to administration of LPS.

The mechanism via which CNPase can regulate neuroinflammation remains unclear. Overproduction of pro-inflammatory mediators by activated microglia may be a risk factor for neurodegenerative onset via the activation of cell-signaling pathways. The nuclear transcriptional factor NF-κB is a key inflammatory regulator. The translocation of NF-κB into the nucleus induces transcription of pro-inflammatory genes by binding to specific promoter regions [43,44]. It is speculated that there might exist an interlink between NF-κB and CNPase. However, NF-κB expression remained relatively unchanged in CNPase silencing cells treated with LPS. It is therefore suggested that increased production of inflammatory cytokines, NO and ROS in CNPase knockdown BV-2 cells due to LPS treatment may be achieved by NF-κB-independent signaling pathways.

ROS are central players in the physiological control of cellular functions as modulators of proinflammatory processes in microglia-associated neurodegenerative diseases [45,46]. Furthermore, a positive feedback loop can be established between ROS and inflammatory cytokines, and these cytokines and chemokines, in turn, stimulate a cascade of events leading to increased oxidative stress via iNOS activation [35]. Studies exploring the mechanisms linking ROS and inflammation found that ROS derived from mitochondria act as signal-transducing molecules that provoke the upregulation of inflammatory cytokine subsets via distinct molecular pathways [47,48]. The present results showed that the production of ROS was significantly increased in activated BV-2 cells with CNPase knockdown, and it has been demonstrated that CNPase expression was mainly localized in the mitochondria. Taken together, it is suggested that CNPase might be involved in the above intricate process either through direct or indirect regulatory mechanism, especially the ROS signal pathway; however, this remains to be further investigated. CNPase has been shown to take part in the regulation of cytoskeleton [49,50], which plays important roles in the cell motility process. In view of this, it was speculated that CNPase might be implicated in the regulation of microglia migration. In the present study, knockdown of CNPase appeared to promote the migration ability of microglia (Additional file 2) indicating that CNPase may inhibit the migration of microglia that may be due to the stabilization of cytoskeletal dynamics, but this awaits further exploration. In addition to this, it has been reported that G1 arrest could be due to ROS generation [51]. Here, we found CNPase knockdown in activated BV-2 cells led to the increased production of ROS coupled by the G1 arrest. This suggests that CNPase may prompt microglia proliferation, as evidenced by the massive increase in activated microglia with highly expressed CNPase in the infarcted zones in MCAO and in the traumatic brain injury areas.

In summary, our results suggest that CNPase might be neuroprotective against microglia-initiated neuroinflammation. Investigation of the possible mechanisms of CNPase in anti-inflammatory roles awaits further investigation. Because CNPase is known to regulate multiple cellular functions, it is important to further explore whether inflammation-related CNPase expression might contribute to disease progression of chronic inflammation in the central nervous system. In this regard, CNPase, which has been shown to take part in the above functions of activated microglial cells, should therefore be considered as a potential therapeutic target among others.

## Conclusions

The present results have provided the first morphological and molecular evidence that CNPase expression is increased in activated microglia. CNPase knockdown resulted in increased expression of various inflammatory mediators. It is concluded that CNPase may play an important role as a putative anti-inflammatory gene both in normal and injured brain.

## Additional files

Additional file 1:
**CNPase expression is mainly localized in mitochondrial protein in BV-2 cells.** Western blot shows the expression of CNPase (48/46 kDa) and β-actin (43 kDa) in mitochondrial (lane 1) and cytosolic protein (lane 2) isolated from the BV-2 microglia respectively. Note that expression of CNPase protein was significantly higher in mitochondria than that in the cytoplasm. Additional file 2:
**Transwell migration assay shows that CNPase knockdown increases the migration of activated BV-2 microglia.** **(A)** Light microscopy images of BV-2 cells transfected with CNPase siRNA or control siRNA in a trans-well chamber. **(B)** The quantitative analysis revealed an increase in the migration of microglia, while the knockdown of CNPase increased the migrating ability of BV-2 microglia. Data are represented as mean ± SD (n = 5), ****P* < 0.001. Scale bar = 1 mm (Aa and Ab) and 100 μm (Ac and Ad).

## Abbreviations

CNPase: 2′,3′-cyclic nucleotide 3′-phosphodiesterase
DMEM: Dulbecco’s modified Eagle’s medium
FBS: fetal bovine serum
FPI: fluid perfusion injury
IL-1β: interleukin-1 beta
iNOS: inducible nitric oxide synthase
LPS: lipopolysaccharide
MCA: middle cerebral artery
MCAO: middle cerebral artery occlusion
NF-κB: nuclear factor kappa-light-chain-enhancer of activated B cells
NO: nitric oxide
PBS: phosphate-buffered saline
PCR: polymerase chain reaction
ROS: reactive oxygen species
RT: reverse transcription
siRNA: small interfering RNA
TNF-α: tumor necrosis factor-alpha

## References

[1] Muller HW, Clapshaw PA, Seifert W (1981). Two molecular forms of the isolated brain enzyme 2′,3′-cyclic nucleotide 3′-phosphodiesterase. FEBS Lett.

[2] Monoh K, Kurihara T, Sakimura K, Takahashi Y (1989). Structure of mouse 2′,3′-cyclic-nucleotide 3′-phosphodiesterase gene. Biochem Biophys Res Commun.

[3] O’Neill RC, Minuk J, Cox ME, Braun PE, Gravel M (1997). CNP2 mRNA directs synthesis of both CNP1 and CNP2 polypeptides. J Neurosci Res.

[4] Gravel M, DeAngelis D, Braun PE (1994). Molecular cloning and characterization of rat brain 2′,3′-cyclic nucleotide 3′-phosphodiesterase isoform 2. J Neurosci Res.

[5] Kurihara T, Monoh K, Sakimura K, Takahashi Y (1990). Alternative splicing of mouse brain 2′,3′-cyclic-nucleotide 3′-phosphodiesterase mRNA. Biochem Biophys Res Commun.

[6] McFerran B, Burgoyne R (1997). 2′,3′-Cyclic nucleotide 3′-phosphodiesterase is associated with mitochondria in diverse adrenal cell types. J Cell Sci.

[7] Brdiczka D, Beutner G, Ruck A, Dolder M, Wallimann T (1998). The molecular structure of mitochondrial contact sites. Their role in regulation of energy metabolism and permeability transition. Biofactors.

[8] Halestrap AP, Brenner C (2003). The adenine nucleotide translocase: a central component of the mitochondrial permeability transition pore and key player in cell death. Curr Med Chem.

[9] Wu CY, Lu J, Cao Q, Guo CH, Gao Q, Ling EA (2006). Expression of 2′,3′-cyclic nucleotide 3′-phosphodiesterase in the amoeboid microglial cells in the developing rat brain. Neuroscience.

[10] Cao Q, Ding P, Lu J, Dheen ST, Moochhala S, Ling EA (2007). 2′,3′-Cyclic nucleotide 3′-phosphodiesterase cells derived from transplanted marrow stromal cells and host tissue contribute to perineurial compartment formation in injured rat spinal cord. J Neurosci Res.

[11] Galvita A, Grachev D, Azarashvili T, Baburina Y, Krestinina O, Stricker R, Reiser G (2009). The brain-specific protein, p42(IP4) (ADAP 1) is localized in mitochondria and involved in regulation of mitochondrial Ca2+. J Neurochem.

[12] Lee J, Gravel M, Zhang R, Thibault P, Braun PE (2005). Process outgrowth in oligodendrocytes is mediated by CNP, a novel microtubule assembly myelin protein. J Cell Biol.

[13] Dreiling CE, Schilling RJ, Reitz RC (1981). 2′,3′-cyclic nucleotide 3′-phosphohydrolase in rat liver mitochondrial membranes. Biochim Biophys Acta.

[14] Azarashvili T, Krestinina O, Galvita A, Grachev D, Baburina Y, Stricker R, Evtodienko Y, Reiser G (2009). Ca2 + −dependent permeability transition regulation in rat brain mitochondria by 2′,3′-cyclic nucleotides and 2′,3′-cyclic nucleotide 3′-phosphodiesterase. Am J Physiol Cell Physiol.

[15] Halestrap AP (2006). Calcium, mitochondria and reperfusion injury: a pore way to die. Biochem Soc Trans.

[16] Dheen ST, Kaur C, Ling EA (2007). Microglial activation and its implications in the brain diseases. Curr Med Chem.

[17] Minagar A, Shapshak P, Fujimura R, Ownby R, Heyes M, Eisdorfer C (2002). The role of macrophage/microglia and astrocytes in the pathogenesis of three neurologic disorders: HIV-associated dementia, Alzheimer disease, and multiple sclerosis. J Neurol Sci.

[18] Kim YS, Joh TH (2006). Microglia, major player in the brain inflammation: their roles in the pathogenesis of Parkinson’s disease. Exp Mol Med.

[19] Tai YF, Pavese N, Gerhard A, Tabrizi SJ, Barker RA, Brooks DJ, Piccini P (2007). Imaging microglial activation in Huntington’s disease. Brain Res Bull.

[20] Colton CA, Gilbert DL (1987). Production of superoxide anions by a CNS macrophage, the microglia. FEBS Lett.

[21] Dheen ST, Jun Y, Yan Z, Tay SS, Ling EA (2005). Retinoic acid inhibits expression of TNF-alpha and iNOS in activated rat microglia. Glia.

[22] Vila M, Jackson-Lewis V, Guegan C, Wu DC, Teismann P, Choi DK, Tieu K, Przedborski S (2001). The role of glial cells in Parkinson’s disease. Curr Opin Neurol.

[23] Wu YP, Ling EA (1998). Transsynaptic changes of neurons and associated microglial reaction in the spinal cord of rats following middle cerebral artery occlusion. Neurosci Lett.

[24] Saura J, Tusell JM, Serratosa J (2003). High-yield isolation of murine microglia by mild trypsinization. Glia.

[25] Nakamura Y, Si QS, Kataoka K (1999). Lipopolysaccharide-induced microglial activation in culture: temporal profiles of morphological change and release of cytokines and nitric oxide. Neurosci Res.

[26] Schmittgen TD, Livak KJ (2008). Analyzing real-time PCR data by the comparative C-T method. Nat Protoc.

[27] Sakamoto Y, Tanaka N, Ichimiya T, Kurihara T, Nakamura KT (2005). Crystal structure of the catalytic fragment of human brain 2′,3′-cyclic-nucleotide 3′-phosphodiesterase. J Mol Biol.

[28] Pelvig DP, Pakkenberg H, Stark AK, Pakkenberg B (2008). Neocortical glial cell numbers in human brains. Neurobiol Aging.

[29] Su AI, Wiltshire T, Batalov S, Lapp H, Ching KA, Block D, Zhang J, Soden R, Hayakawa M, Kreiman G, Cooke MP, Walker JR, Hogenesch JB (2004). A gene atlas of the mouse and human protein-encoding transcriptomes. Proc Natl Acad Sci U S A.

[30] Vogel US, Thompson RJ (1988). Molecular structure, localization, and possible functions of the myelin-associated enzyme 2′,3′-cyclic nucleotide 3′-phosphodiesterase. J Neurochem.

[31] Sprinkle TJ, Agee JF, Tippins RB, Chamberlain CR, Faguet GB, De Vries GH (1987). Monoclonal antibody production to human and bovine 2′,3′-cyclic nucleotide 3′-phosphodiesterase (CNPase): high-specificity recognition in whole brain acetone powders and conservation of sequence between CNP1 and CNP2. Brain Res.

[32] Lee J, O’Neill RC, Park MW, Gravel M, Braun PE (2006). Mitochondrial localization of CNP2 is regulated by phosphorylation of the N-terminal targeting signal by PKC: implications of a mitochondrial function for CNP2 in glial and non-glial cells. Mol Cell Neurosci.

[33] Kreutzberg GW (1996). Microglia: a sensor for pathological events in the CNS. Trends Neurosci.

[34] Ziv Y, Ron N, Butovsky O, Landa G, Sudai E, Greenberg N, Cohen H, Kipnis J, Schwartz M (2006). Immune cells contribute to the maintenance of neurogenesis and spatial learning abilities in adulthood. Nat Neurosci.

[35] Urrutia PJ, Mena NP, Nunez MT (2014). The interplay between iron accumulation, mitochondrial dysfunction, and inflammation during the execution step of neurodegenerative disorders. Front Pharmacol.

[36] Koizumi S, Ohsawa K, Inoue K, Kohsaka S (2013). Purinergic receptors in microglia: functional modal shifts of microglia mediated by P2 and P1 receptors. Glia.

[37] Dibaj P, Nadrigny F, Steffens H, Scheller A, Hirrlinger J, Schomburg ED, Neusch C, Kirchhoff F (2010). NO mediates microglial response to acute spinal cord injury under ATP control in vivo. Glia.

[38] Popa C, Netea MG, van Riel PL, van der Meer JW, Stalenhoef AF (2007). The role of TNF-alpha in chronic inflammatory conditions, intermediary metabolism, and cardiovascular risk. J Lipid Res.

[39] Takeuchi H, Jin S, Wang J, Zhang G, Kawanokuchi J, Kuno R, Sonobe Y, Mizuno T, Suzumura A (2006). Tumor necrosis factor-alpha induces neurotoxicity via glutamate release from hemichannels of activated microglia in an autocrine manner. J Biol Chem.

[40] Ohshima H, Bartsch H (1994). Chronic infections and inflammatory processes as cancer risk factors: possible role of nitric oxide in carcinogenesis. Mutat Res.

[41] Fujii T, Iwane AH, Yanagida T, Namba K (2010). Direct visualization of secondary structures of F-actin by electron cryomicroscopy. Nature.

[42] Mullins RD, Hansen SD (2013). In vitro studies of actin filament and network dynamics. Curr Opin Cell Biol.

[43] Baima ET, Guzova JA, Mathialagan S, Nagiec EE, Hardy MM, Song LR, Bonar SL, Weinberg RA, Selness SR, Woodard SS, Chrencik J, Hood WF, Schindler JF, Kishore N, Mbalaviele G (2010). Novel insights into the cellular mechanisms of the anti-inflammatory effects of NF-kappaB essential modulator binding domain peptides. J Biol Chem.

[44] Lee JW, Lee MS, Kim TH, Lee HJ, Hong SS, Noh YH, Hwang BY, Ro JS, Hong JT (2007). Inhibitory effect of inflexinol on nitric oxide generation and iNOS expression via inhibition of NF-kappaB activation. Mediators Inflamm.

[45] Dokic I, Hartmann C, Herold-Mende C, Regnier-Vigouroux A (2012). Glutathione peroxidase 1 activity dictates the sensitivity of glioblastoma cells to oxidative stress. Glia.

[46] Shen SC, Wu MS, Lin HY, Yang LY, Chen YH, Chen YC: **Reactive oxygen species-dependent nitric oxide production in reciprocal interactions of glioma and microglial cells.** *J Cell Physiol* 2014. doi: 10.1002/jcp.24659. 10.1002/jcp.24659 24777714

[47] Naik E, Dixit VM (2011). Mitochondrial reactive oxygen species drive proinflammatory cytokine production. J Exp Med.

[48] Tschopp J (2011). Mitochondria: sovereign of inflammation?. Eur J Immunol.

[49] Block ML, Zecca L, Hong JS (2007). Microglia-mediated neurotoxicity: uncovering the molecular mechanisms. Nat Rev Neurosci.

[50] Brown GC, Bal-Price A (2003). Inflammatory neurodegeneration mediated by nitric oxide, glutamate, and mitochondria. Mol Neurobiol.

[51] Park GB, Bang SR, Lee HK, Kim D, Kim S, Kim JK, Kim YS, Hur DY (2014). Ligation of CD47 induces G1 arrest in EBV-transformed B cells through ROS generation, p38 MAPK/JNK activation, and Tap73 upregulation. J Immunother.

