# Supplementary figures and images for: Expression of 2′,3′-cyclic nucleotide 3′-phosphodiesterase (CNPase) and its roles in activated microglia in vivo and in vitro
Source: J Neuroinflammation. 2014 Aug 23;11:148. doi: 10.1186/s12974-014-0148-9 (PMC4244045; doi:10.1186/s12974-014-0148-9)

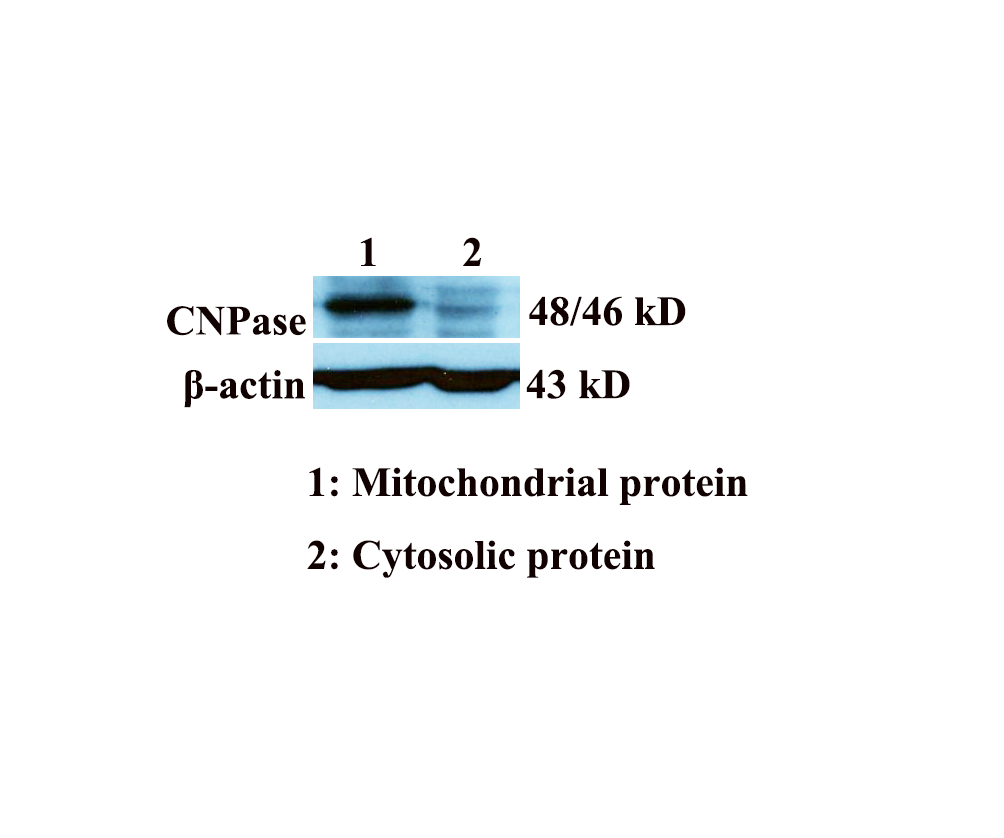

Supplement: Additional file 1: — CNPase expression is mainly localized in mitochondrial protein in BV-2 cells. Western blot shows the expression of CNPase (48/46 kDa) and β-actin (43 kDa) in mitochondrial (lane 1) and cytosolic protein (lane 2) isolated from the BV-2 microglia respectively. Note that expression of CNPase protein was significantly higher in mitochondria than that in the cytoplasm. [file 12974_2014_148_MOESM1_ESM.tiff]

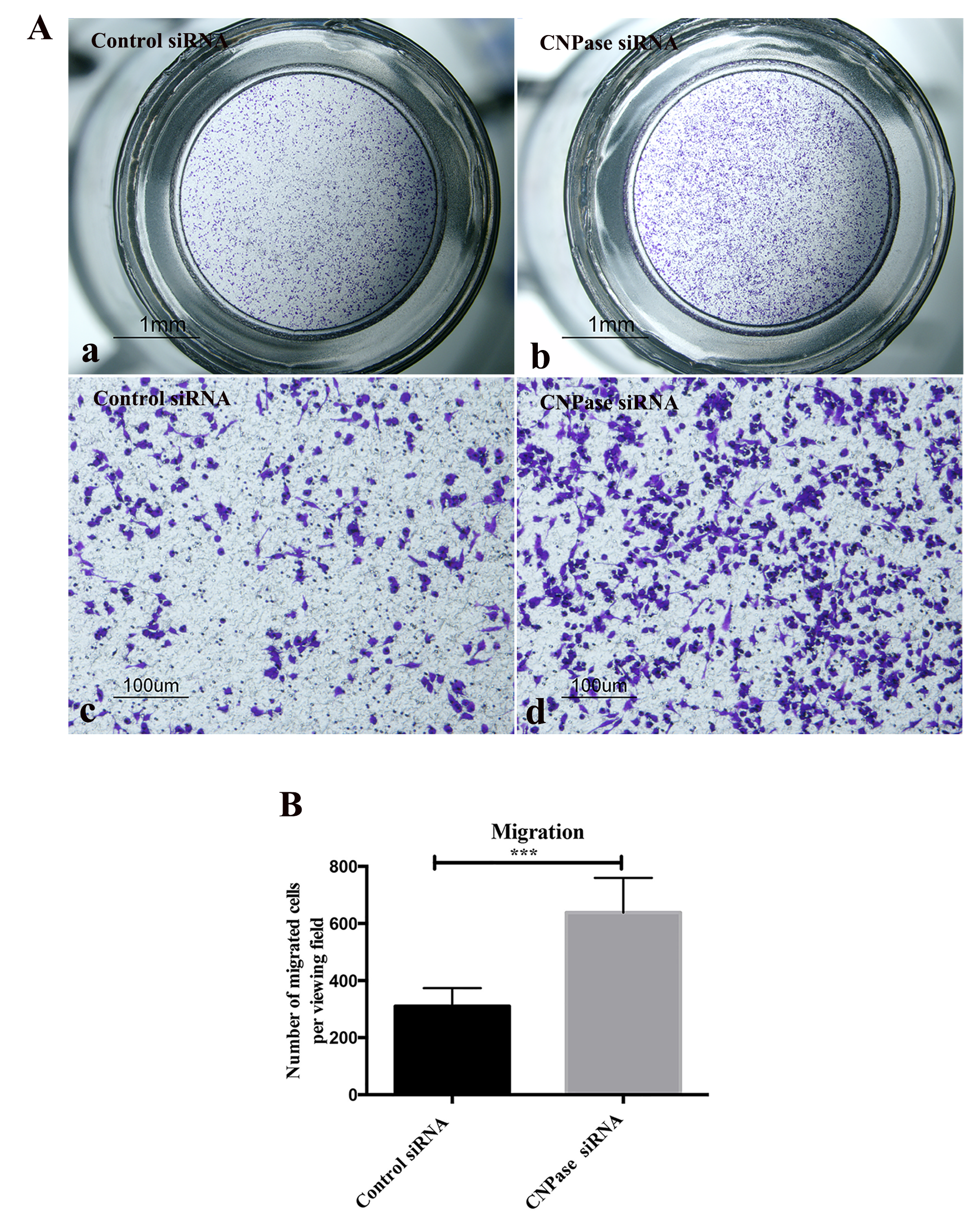

Supplement: Additional file 2: — Transwell migration assay shows that CNPase knockdown increases the migration of activated BV-2 microglia. (A) Light microscopy images of BV-2 cells transfected with CNPase siRNA or control siRNA in a trans-well chamber. (B) The quantitative analysis revealed an increase in the migration of microglia, while the knockdown of CNPase increased the migrating ability of BV-2 microglia. Data are represented as mean ± SD (n = 5), ***P < 0.001. Scale bar = 1 mm (Aa and Ab) and 100 μm (Ac and Ad). [file 12974_2014_148_MOESM2_ESM.tiff]
